# Structural and developmental characteristics of Japanese elite athletes: a longitudinal case study of a talent identification and development programme in Fukuoka prefecture

**DOI:** 10.3389/fspor.2026.1748585

**Published:** 2026-02-09

**Authors:** Masahiro Hagiwara, Taisuke Kinugasa, Miki Haramura, Takehira Nakao, Kazuto Teshima, Shuhei Yamashita, Katsuyoshi Shirai

**Affiliations:** 1 Japan Institute of Sports Sciences, Japan High Performance Sport Center, Japan Sport Council, Tokyo, Japan; 2 The Center for Liberal Arts, Meiji Gakuin University, Kanagawa, Japan; 3 Faculty of Human Science, Kyushu Sangyo University, Fukuoka, Japan; 4 Fukuoka Prefecture Institute of Sports Science and Information, Fukuoka Prefecture Sports Council, Fukuoka, Japan

**Keywords:** anthropometry, athlete development pathways, developmental environment, physical fitness, relative age effect, sex difference, talent transfer, sporting history

## Abstract

**Purpose:**

This study longitudinally examined the influence of sporting history, the relative age effect (RAE), and physical development on the elite progression of Japanese athletes from a local talent identification and development (L-TID) programme to senior elite status.

**Methods:**

Data were collected from 383 L-TID graduates (31 senior elite athletes and 352 talented athletes). The cohort demonstrated significantly superior physical fitness and consistent physical advantages. Logistic regression analysis was conducted across 5 academic years (from 5th grade in elementary school to 9th grade in junior high school) to identify consistent predictors of elite status.

**Results:**

Senior elite athletes were highly concentrated in a limited number of late-specialisation sports, with 90% having experienced a sports transition. Sex (female) and sports transitioning emerged as the most consistent predictors of elite attainment across all grades. While talented athletes initially demonstrated significant RAE and superior physical fitness upon entry, these advantages diminished by the senior elite level. General anthropometric and fitness measures (except body mass in grades 8–9) were not reliable predictors of long-term elite progression.

**Conclusion:**

Elite attainment within the L-TID programme, targeting a cohort with superior athletic ability, is predominantly influenced by structural and developmental factors (female participation and late specialisation) rather than by initial RAE or physical attributes. These findings support talent development strategies that foster diverse sporting experiences and deliberate talent transfer, highlighting a shift away from early RAE and physical-based selection towards more holistic and adaptive developmental approaches reflecting the Japanese sporting and social context.

## 1 Introduction

Effective talent identification and development (TID) programmes are widely recognised as foundational components of successful high-performance sports systems globally (1, 2). Over the past few decades, many countries have invested in structured athlete development pathways aimed at detecting and nurturing athletic potential from an early age (2, 3). These systems have increasingly emphasised multidimensional approaches to TID (incorporating physical, psychological, and sociological factors), alongside long-term developmental planning and adaptability across diverse sporting contexts (2–4).

Despite these advances, a main challenge persists insufficiently addressed that there is a critical lack of empirical evidence evaluating the long-term, real-world effectiveness of TID at a system level. Specifically, limited evidence has identified which early developmental trajectories most reliably lead to sustained elite performance. This knowledge gap impairs the ability of policymakers and practitioners to design systems that maximise long-term outcomes. Addressing this issue is particularly relevant in comprehensive national systems like Japan's local TID (L-TID) programme, where system-level decisions influence of developing athletes. Thus, this study directly responds to the need for evidence-based models of athlete development pathways by investigating which early-stage factors are most predictive of elite attainment.

In Japan, recent government initiatives have called improved international competitiveness by optimising resource allocation at the local (prefectural) level (5, 6). Among these efforts, Fukuoka Prefecture launched a pioneering L-TID programme in 2004, supported by national governing bodies such as the Japan Sport Council and the Japanese Olympic Committee (7). This programme focuses on talent detection by matching children to sport that suit their physical aptitudes, regardless of prior sporting experience. Participants are exposed to approximately 10 sports through multi-sports training including athletics, fencing, and shooting. Key feature of this model include emphasis on early multi-sport exposure, sport matching (i.e., an alignment of individuals to sports based on aptitude), and sports transitioning (i.e., reallocating athletes to more suitable sports following initial exposure and sport matching) (7, 8). These features distinguish the Japanese system from many Western TID approaches, which tend to prioritise talent transfer at a later stage in athlete development pathways (4, 9).

Since its inception, the Fukuoka L-TID initiative has expanded nationwide, with approximately 50 local governments implementing similar L-TID programmes that vary in scale and design based on their available resources (10). A total of 24 Olympians, including gold medalists, have emerged from the L-TID graduates across Japan (Tokyo 2020: 11; Beijing 2022: 5; Paris 2024: 13). These outcomes gradually demonstrate the programme's efficacy and have garnered significant attention not only from L-TID stakeholders but also from experts in high performance sports area across Japan. Structurally, the L-TID programmes are primarily target elementary and junior high school students and typically rely on physical fitness testing for initial selection. Many of the TID models since aligned with the Japanese FTEM (Foundation, Talent, Elite, and Mastery) framework, which outlines progressive structure for athlete development pathways at the system level (11).

The present study focuses on the foundational Fukuoka L-TID model which is a unique case for longitudinal analysis. The programme has been operational for 20 years, has pioneered multi-sport design, has consistently received governmental support, and has a proven track record of producing eight Olympians, including two medalists. These factors make it an unparalleled setting for conducting robust, longitudinal research into the determinants of success within the Japanese L-TID programme.

Despite growing adoption and policy support, empirical studies evaluating the long-term effectiveness of L-TID programmes remains limited. Previous research has highlighted key developmental factors, such as deliberate play, multi-sport participation, structured practice and competition in other sports, deliberate practice in the main sport, and strategic talent transfer initiatives as influential in athlete development (8). Other studies have focused on the relative age effect (RAE), which is a developmental advantage linked to birth month within an age group, or compared the physical attributes of TID-selected athletes with their non-selected peers (12–14). However, three critical limitations remain in the literature. First, few studies have longitudinally examined the impact of sports transitioning on long-term outcomes, especially within structured TID systems. Second, while RAE is well documented, its long-term influence on elite attainment remains unclear. Third, there is limited evidence regarding the persistence of early physical advantages into adulthood. Importantly, no existing studies integrated these developmental dimensions to explain successful transition from early TID to senior elite status. This gap necessitates a multi-faceted, longitudinal approach. Therefore, the present study focuses on (1) sporting history and transitioning, (2) the Relative Age Effect (RAE), and (3) physical development, as these are the key but insufficiently integrated factors determining long-term athletic success in real-world TID settings.

To our knowledge, this study is the first study to longitudinally integrate multi-dimensional data within a government-supported TID system over a 20-year period. It addresses these gaps by first examining anthropometric and physical fitness differences and then analysing the developmental trajectories that differentiating athletes who progressed to the elite level. The novelty of this study lies in its application of the Japanese FTEM framework to an unprecedented 20 years of historical data from Japan's first and longest-running L-TID programme. This approach generates evidence-based insights into the long-term outcomes of early-stage TID and informs the design of more effective and inclusive athlete development systems in both Japan and broader international contexts.

## 2 Materials and methods

### 2.1 The core concept of the Fukuoka L-TID model

The core concept of the Fukuoka L-TID model revolves around the “Identification,” “Development,” and “Matching” of athletic potential among primary and secondary school students in Fukuoka Prefecture (15). The programme begins by providing all participants with detailed feedback based on physical fitness test results to promote future skill development and suggest suitable sports aligned with individual strengths. To maximise the opportunity for selected participants to progress to higher levels, they engage in a specialised Physical Ability Development Programme. The programme aims to cultivate athletic potential and develop the skills needed for high-level competition through three main components: the Physical Ability Development Programme (focuses on developing coordination and movement skills through diverse sport experiences including rugby, fencing, and judo), the Intellectual Ability Development Programme (enhances communication skills and logical thinking through lectures on topics such as sports nutrition and mental training), and the Parent Support Programme (equips parents with knowledge in nutrition and sports science to support the athlete's daily life). This comprehensive, multi-dimensional approach, developed in cooperation with various sports organisations, enables athletes to explore a wide range of sporting experiences and ultimately identify the sport that best matches their individual potential.

The Fukuoka L-TID cohort, similar to other Japanese L-TID initiatives, is composed of individuals who exhibit superior athletic ability compared to age-matched Japanese general peers (13, 14). This group is determined through a rigorous three-stage selection process, with physical fitness assessments integrated into each round. Considering that the number of applicants for the initial selection round has sometimes exceeded 50,000, from which approximately 30 athletes are ultimately selected (16), this process clearly functions as a highly competitive and advanced screening for athletic potential (Table 1).

**Table 1:** Current, process and progression of participants in the Fukuoka TID programme (2024–2025 cycle example).

Selection Step	Number of applicants/Successful candidates
First selection	56,817 applicants in 2024 (16)
↓	
Second selection	No official data available
↓	
Third selection	No official data available
↓	
TID programme participants in 2025 (Successful candidates of third selection)	Approximately 30 athletes per grade level (16)
	Elementary school:
	Grade 5: 26 participants (11 Male, 15 Female)
	Grade 6: 35 participants (19 Male, 16 Female)
	Junior high school:
	Grade 7: 32 participants (16 Male, 16 Female)
	Grade 8: 34 participants (21 Male, 13 Female)
	Grade 9: 31 participants (19 Male, 12 Female)

Note, Physical fitness tests screen for participants with superior athletic ability in all selection steps.

### 2.2 Subjects

Between 2005 and 2019, a total of 383 athletes (182 males and 201 females) graduated from Fukuoka L-TID programme. Among them, 31 (6 males, 25 females) advanced to the Elite stage by representing Japan at the senior level (defined as “elite athletes”), while the remaining 352 (176 males, 176 females) participated only at the Talent stage as “talented athletes” in accordance with the Japanese FTEM framework (11). Elite achievement was attained by 3.3% of male participants and 12.4% of female participants. The study received ethics approval from the first author's Institute Ethics Committee (Approval Number: 2022-012) and was conducted in accordance with the Declaration of Helsinki.

### 2.3 Study design

Initially, the anthropometric and physical fitness indices of the subjects, using measures comparable to the national standard, were compared with those of Japanese age-matched general peers to verify their level. Subsequently, this study examined the characteristics of athletes during elementary and junior high school at two developmental stages (i.e., Talent and Elite), focusing on the following aspects: (1) sporting history and sports transitioning; (2) RAE distribution by birth quarter; (3) anthropometric and physical fitness development.

### 2.4 Sporting history and sports transitioning

Participants' sporting histories from elementary through high school were systematically recorded. The final main sport was defined as the most recently recorded sport in which the participants was actively engaged. Sports transitioning was categorised based on continuity between early and later sport participation: individuals were classified as having “continued in the same sport” if the sport engaged in during elementary school matched their final main sport, and as having “transitioned to a new sport” if these differed. Cases where early sport participation data was unavailable or unclear were categorized as “unconfirmed”.

### 2.5 RAE distribution by birth quarter

The birth dates were recorded at the time of L-TID application, when they were in the 4th grade of elementary school. Birth months were grouped based on the Japanese fiscal year (Q1: April to May, Q2: June to September, Q3: October to December, and Q4: January to March), following Nakata & Sakamoto (17, 18) and Sasano et al. (19).

### 2.6 Anthropometric and physical fitness development

Physical and fitness measures were collected from 5th grade of elementary school to 3rd year of junior high school (9th grade), including height, body mass, 25-m sprint, standing broad jump, standing quintuple jump, rebound drop-jump, reaction time, and 20-m shuttle run. The testing were conducted in strict adherence to national standard protocols, the MEXT's New Physical Fitness Test Guidelines (20), were used for height, body mass, standing broad jump, and 20-m shuttle run. Validated protocols were used for the remaining measures (21–23). Trained physical education teachers and the programme graduates administrated all the measures under supervision to ensure standardisation. Sample sizes varied by measure, as not all athletes completed every test.

### 2.7 Statistical analysis

Hedges' *g* (24) was calculated to determine the effect sizes (*ES*) for differences between all subjects (entire cohort) and age-matched Japanese general peers (25). The resulting *ES* magnitudes were interpreted using Cohen's criteria (26), defining “medium” effects as 0.5 ≤ |*g*| < 0.8 and “large” effects as |*g*| ≥ 0.8. Frequencies and proportions were calculated for sporting history. Birth quarter distributions were compared against even distribution using a *χ*^2^ goodness-of-fit test. Odds Ratios (*OR*) and 95% Confidence Intervals (*95% CI*) were computed. To identify *OR*s differentiating elite athletes (E) from talented athletes (T), logistic regression was conducted for each grade. The dependent variable was the elite status (Elite = 1, Talented = 0). Independent variables included: Sex (Female = 1, Men = 0), Sports transitioning (Yes = 1, No = 0), Birth month (Early = Q1 and Q2 = 1, Late = Q3 and Q4 = 0), Sport type (Individual = 1, Team = 0), Height, Body mass, 25-m sprint, Standing broad jump, Standing quintuple jump, Rebound drop-jump, Reaction time, and 20-m shuttle run. A forward stepwise selection method was employed to construct the most appropriate model for each grade level. The model goodness-of-fit was assessed using the *Nagelkerke R*^2^ and Chi-square (*χ*^2^) statistics. The significance of individual predictors was evaluated using the *Wald* statistic. Results are presented as *ORs* with their corresponding *95% CI*. Statistical significance was set at *P* < .05. The various statistical values were interpreted with references to previous research (26, 27).

## 3 Results

Tables 2, 3 present the results of the comparison of anthropometric and physical fitness indices between all subjects (entire cohort) and age-matched Japanese general peers. The subject group consistently showed greater average anthropometric measures than their peers. Notably, the superior in height was supported by medium or large effect sizes (Hedges' |*g*| ≥ 0.5) across most grades and both sexes. Furthermore, all subjects demonstrated significantly superior performance in both the standing broad jump and the 20-m shuttle run compared to their general peers across all grades and both sexes with large effect sizes (Hedges' |*g*| ≥ 0.8).

**Table 2:** Comparison of anthropometric between all subjects and age-matched general peers across from 5th grade elementary to the 9th grade junior high school in Fukuoka talent identification and development programme.

Grade		Sex	Height (cm)						Body mass (kg)					
			All subjects		vs.		Age-matched general peers (25)		All subjects		vs.		Age-matched general peers (25)	
			*n*	*Mean* ± *SD*	*ES*		*n*	*Mean* ± *SD*	*n*	*Mean* ± *SD*	*ES*		*n*	*Mean* ± *SD*
Elementary school	5	Male	141	145.0 ± 7.0	0.83	*gg*	769	140.0 ± 5.9	132	36.6 ± 5.6	0.36		734	34.4 ± 6.2
		Female	169	144.9 ± 6.5	0.51	*g*	567	141.5 ± 6.9	161	35.5 ± 5.5	0.17		551	34.5 ± 6.1
	6	Male	148	152.9 ± 8.3	0.91	*gg*	749	146.1 ± 7.4	148	42.5 ± 7.7	0.52	*g*	734	38.7 ± 7.2
		Female	177	151.1 ± 6.3	0.46		530	148.1 ± 6.6	177	40.7 ± 6.1	0.18		517	39.4 ± 7.1
Junior high school	7	Male	169	160.4 ± 7.6	0.75	*g*	1,114	154.6 ± 7.9	169	48.9 ± 8.3	0.46		1,088	44.6 ± 9.5
		Female	189	155.8 ± 5.2	0.57	*g*	861	152.8 ± 5.3	189	45.5 ± 5.7	0.24		841	43.9 ± 6.9
	8	Male	175	166.1 ± 6.1	0.54	*g*	1,186	162.2 ± 7.4	176	54.6 ± 7.5	0.53	*g*	1,162	50.2 ± 8.3
		Female	191	158.2 ± 4.9	0.45		898	155.9 ± 5.3	191	48.9 ± 5.6	0.23		882	47.4 ± 6.6
	9	Male	176	169.3 ± 5.5	0.46		1,125	166.4 ± 6.5	176	59.2 ± 7.1	0.64	*g*	1,101	54.2 ± 8.0
		Female	190	159.5 ± 4.9	0.56	*g*	873	156.7 ± 5.1	190	51.5 ± 5.5	0.37		857	49.4 ± 5.9

Note, Data are shown as *Mean* ± *SD*. Determination of effect size (*ES*) according to Hedge's *g* (All subjects vs. Age-matched general peers), *g*: *ES* was medium (0.5 ≤ |*g*| < 0.8), *gg*: *ES* was large (*g* > 0.8), Age-matched general peers: Japanese general peers who belonging to a sports club.

**Table 3:** Comparison of physical fitness between all subjects and age-matched general peers across from 5th grade elementary to the 9th grade junior high school in Fukuoka talent identification and development programme.

Grade		Sex	Standing broad jump (cm)						20-m shuttle run (times)					
			All subjects		vs.		Age-matched general peers (25)		All subjects		vs.		Age-matched general peers (25)	
			*n*	*Mean* ± *SD*	*ES*		*n*	*Mean* ± *SD*	*n*	*Mean* ± *SD*	*ES*		*n*	*Mean* ± *SD*
Elementary school	5	Male	59	199.5 ± 12.2	2.12	*gg*	770	157.8 ± 20.1	49	91 ± 11	1.68	*gg*	774	56 ± 21
		Female	93	189.8 ± 10.8	2.00	*gg*	568	152.5 ± 19.6	72	78 ± 14	1.99	*gg*	563	46 ± 17
	6	Male	97	211.0 ± 14.7	2.02	*gg*	755	169.6 ± 21.1	98	96 ± 13	1.46	*gg*	748	66 ± 22
		Female	130	198.9 ± 13.0	2.01	*gg*	530	161.6 ± 19.8	131	85 ± 14	1.82	*gg*	526	53 ± 18
Junior high school	7	Male	149	225.4 ± 16.2	1.62	*gg*	1,119	186.7 ± 24.6	143	108 ± 14	1.63	*gg*	914	72 ± 24
		Female	169	206.0 ± 11.9	1.70	*gg*	853	171.5 ± 21.6	167	92 ± 15	2.07	*gg*	695	53 ± 19
	8	Male	168	238.4 ± 15.2	1.42	*gg*	1,179	204.8 ± 24.8	163	117 ± 14	1.46	*gg*	966	85 ± 22
		Female	187	208.9 ± 12.1	1.55	*gg*	900	177.6 ± 21.4	182	93 ± 16	1.70	*gg*	740	61 ± 20
	9	Male	175	247.7 ± 14.9	1.29	gg	1,128	218.9 ± 23.4	168	115 ± 18	0.92	*gg*	920	95 ± 22
		Female	188	210.9 ± 12.9	1.53	gg	869	180.5 ± 21.1	185	89 ± 16	1.58	*gg*	695	62 ± 18

Note, Data are shown as *Mean* ± *SD*. Determination of effect size (*ES*) according to Hedge's *g* (All subjects vs. Age-matched general peers), *g*: *ES* was medium (0.5 ≤ |*g*| < 0.8), *gg*: *ES* was large (*g* > 0.8), Age-matched general peers: Japanese general peers who belonging to a sports club.

Tables 4, 5 show the final main sports (disciplines) chosen by elite and talented athletes, respectively. A smaller and more concentrated selection of sports was observed among elite athletes (13 sports in total) compared to talented athletes (42 sports). Moreover, the distribution of athletes across these sports was unevenly.

**Table 4:** Final main sports of elite athletes in Fukuoka talent identification and development programme.

Sport disciplines	Male	Female	Total
Fencing	1	9	10
Short Track Speed Skating	1	3	4
Track Cycling	0	3	3
Athletics (Throwing events)	1	1	2
Rowing	0	2	2
Rugby	0	2	2
Shooting	0	2	2
Athletics (Jumping events)	1	0	1
Football	1	0	1
Freestyle Skiing	0	1	1
Handball	1	0	1
Modern Pentathlon	0	1	1
Sailing	0	1	1
Total (All 13 sports disciplines)	6	25	31

**Table 5:** Final main sports of talented athletes in Fukuoka talent identification and development programme.

Sport disciplines	Male	Female	Total
Athletics (Sprint events)	25	11	36
Fencing	12	13	25
Basketball	7	16	23
Baseball	21	1	22
Rugby	15	6	21
Archery	7	11	18
Athletics (Jumping events)	8	10	18
Football	9	8	17
Hockey	6	9	15
Handball	10	4	14
Athletics (Middle/Long distance events)	7	6	13
Shooting	3	10	13
Volleyball	2	9	11
Athletics (Throwing events)	3	7	10
Softball	0	10	10
Weightlifting	6	4	10
Sailing	5	3	8
Wrestling	5	3	8
Rowing	2	4	6
Boxing	4	2	6
Cycling	3	3	6
Judo	1	4	5
Kendo	3	2	5
Swimming	3	1	4
Athletics (Combined events)	0	3	3
Athletics (Sprint and Jumping events)	1	1	2
Badminton	1	1	2
Canoeing	1	1	2
Golf	1	1	2
Powerboating	0	2	2
Short Track Speed Skating	1	1	2
Sport Climbing	1	1	2
Tennis	0	2	2
Triathlon	0	1	1
American Football	1	0	1
Figure Skating	0	1	1
Ice Hockey	1	0	1
Kyudo	0	1	1
Modern Pentathlon	1	0	1
Skiing	0	1	1
Soft Tennis	0	1	1
Ultimate	0	1	1
Total (All 42 sports disciplines)	176	176	352

Table 6 shows sports transitioning and continuation trends. A higher proportion of elite athletes (90.0%) had experience sports transitioning, compared to talented athletes (58.2%).

**Table 6:** Sports transitioning among elite and talented athletes in Fukuoka talent identification and development programme.

Sports transitioning status	Elite athletes						Talented athletes					
	Male		Female		Total		Male		Female		Total	
	*n*	(%)	*n*	(%)	*n*	(%)	*n*	(%)	*n*	(%)	*n*	(%)
Transitioned to a new sport	4	(66.7)	24	(95.8)	28	(90.0)	103	(58.5)	102	(58.0)	205	(58.2)
Continued in the same sport	2	(33.3)	1	(4.2)	3	(10.0)	66	(37.5)	69	(39.2)	135	(38.4)
Unconfirmed	0	(0.0)	0	(0.0)	0	(0.0)	7	(4.0)	5	(2.8)	12	(3.4)
Total	6	(100.0)	25	(100.0)	31	(100.0)	176	(100.0)	176	(100.0)	352	(100.0)

Table 7 presents the RAE patterns. Among talented athletes, both male and female participants demonstrated a significant RAE, with the highest birth distribution in Q1 (Apr-Jun) and the lowest in Q4 (Jan-Mar) (*P* < .001). In contrast, no significant RAE was detected among elite athletes (*P* = .125).

**Table 7:** Relative age effect among talented and elite athletes in Fukuoka talent identification and development programme.

Category		Q1		Q2		Q3		Q4		Total		*χ^2^*	*df*	*P*	*OR*	*95%CI*	
		(Apr-Jun)		(Jul-Sep)		(Oct-Dec)		(Jan-Mar)									
		*n*	(%)	*n*	(%)	*n*	(%)	*n*	(%)	*n*	(%)						
Talented athletes	Male	91	(51.7)	42	(23.9)	31	(17.6)	12	(6.8)	176	(100.0)	77.409	3	<.001***	14.63	7.59	28.21
	Female	78	(44.3)	42	(23.9)	42	(23.9)	14	(8.0)	176	(100.0)	46.909	3	<.001***	9.04	4.85	16.84
Elite athletes	Male & Female	13	(41.9)	8	(25.8)	4	(12.9)	6	(19.4)	31	(100.0)	5.774	3	0.123	3.01	0.96	9.42

Note, Elite athletes: representing Japan at the senior level from Fukuoka talent identification and development programme. Talented athletes: identified through Fukuoka talent identification and development programme.

Odds ratio (*OR*) and 95% confidence interval (*CI*) were calculated for the ratio of Q1 to Q4.

*** *P* < .001.

Table 8 reports the results of logistic regression analysis by academic grade level. The overall models for all grades (from 5th to 9th grade) were statistically significant (all *χ^2^*, *P* < .001). The *Nagelkerke R^2^* values ranged from .168 (7th grade) to .391 (5th grade), indicating varying degrees of explanatory power across the models. Significant predictors identified in most models included Sex (Female = 1), Sports transitioning (Yes = 1), and 20-m shuttle run score. Body mass was also identified as a significant predictor in both 8th and 9th grades. However, other anthropometric and physical fitness measures did not significantly predict elite status. Sex was identified as a consistent predictor across all grades; however, it should be noted that the exceptionally high *OR*s for sex (e.g., *OR* = 127.696, *P* < .001) likely reflect statistical instability due to the small elite sample size and cohort imbalance. Therefore, these values should be interpreted as programme-specific observations rather than generalized developmental advantages. Sports transitioning was also a significant factor in all grades with *OR*s ranging from 5.867 (7th grade, *P* = .006) to 27.047 (5th grade, *P* = .005). The 20-m shuttle run score showed significant positive influence in all grades (all *P* ≤ .020), with corresponding *OR*s (e.g., *OR* = 1.092 in 5th grade, *OR* = 1.069 in 9th grade). Body mass was a significant positive predictor only in 8th grade of junior high school (*OR* = 1.135, *P* = 0.011*)* and 9th grade (*OR* = 1.160, *P* < .001). This suggests that a one-unit increase in body mass was associated with a 13.5%–16.0% increase in the odds of being an elite athlete in these grades, holding other factors constant.

**Table 8:** Results of logistic regression analysis on factors differentiating elite athletes across from 5th grade elementary to the 9th grade junior high school in Fukuoka talent identification and development programme.

Grade		Model coefficients				Variables in the equation								
		n	χ^2^	*P*	Nagelkerke *R*^2^	Variable	*Coefficient*	*SE*	*Wald*	*df*	*P*	*OR*	*95% CI*	
		(T, E)	(*df*)											
Elementary school	5	109	26.448	<.001***	0.391	Sex (Female)	3.051	0.988	9.543	1	.002**	21.138	3.050	146.475
		(94, 15)	(3)			Sports transitioning (Yes)	3.298	1.177	7.856	1	.005**	27.047	2.696	271.396
						20m-shuttle run	0.088	0.032	7.669	1	.006**	1.092	1.026	1.162
						(Constant)	−14.106	3.727	14.322	1	<.001***	0.000		
	6	199	26.751	<.001***	0.246	Sex (Female)	2.062	0.616	11.191	1	<.001***	7.861	2.349	26.310
		(176, 23)	(3)			Sports transitioning (Yes)	1.966	0.679	8.383	1	.003**	7.140	1.887	27.013
						20m-shuttle run	0.065	0.019	11.532	1	<.001***	1.068	1.028	1.109
						(Constant)	−10.942	2.267	23.287	1	<.001***	0.000		
Junior high school	7	301	22.455	<.001***	0.168	Sex (Female)	2.020	0.591	11.675	1	<.001***	7.535	2.366	24.001
		(277, 24)	(3)			Sports transitioning (Yes)	1.769	0.642	7.591	1	.006**	5.867	1.666	20.654
						20m-shuttle run	0.035	0.015	5.412	1	.020*	1.036	1.006	1.067
						(Constant)	−8.608	1.918	20.132	1	<.001***	0.000		
	8	333	30.690	<.001***	0.235	Sex (Female)	3.876	0.927	17.478	1	<.001***	48.219	7.836	296.722
		(311, 21)	(4)			Sports transitioning (Yes)	1.818	0.669	7.380	1	.007**	6.158	1.659	22.854
						Body mass	0.127	0.050	6.492	1	.011*	1.135	1.030	1.252
						20m-shuttle run	0.056	0.018	10.045	1	.002**	1.057	1.021	1.094
						(Constant)	−19.053	4.470	18.173	1	<.001***	0.000		
	9	333	42.745	<.001***	0.312	Sex (Female)	4.850	1.005	23.277	1	<.001***	127.696	17.806	915.783
		(312, 22)	(4)			Sports transitioning (Yes)	2.389	0.796	8.998	1	.003**	10.900	2.289	51.913
						Body mass	0.148	0.045	10.947	1	<.001***	1.160	1.062	1.266
						20m-shuttle run	0.067	0.016	16.564	1	<.001***	1.069	1.035	1.104
						(Constant)	−22.924	4.467	26.339	1	<.001***	0.000		

Note, A forward stepwise selection method was used for the logistic regression analysis. Grade refers to the academic year from elementary school entrance to junior high school graduation., T, Talented athletes; E, Elite athletes; *df*, Degrees of freedom; *P*, *P*-value; *OR*, Odd ratiol *SE*, Standard error; *CI,*: confidence interval; Sex (Female = 1, Male = 0), Sports transitioning (Yes = 1, No = 0).

* *P* < .05.

** *P* < .01.

*** *P* < .001.

## 4 Discussion

The present study yielded several key findings regarding the characteristics that distinguish athletes who transition from a Japanese L-TID programme to senior elite status. It was initially confirmed that the entire cohort in this study exhibited superior anthropometric and physical fitness attributes (Tables 2, 3), consistent with previous research (13, 14). The primary findings within the context of this L-TID programme and its target cohort are presented below. First, elite athletes tended to concentrate in a small number of late-specialisation sports (e.g., athletics, cycling, rowing), and most had experience sports transitioning (Tables 4–6). Second, while a significant RAE was observed among talented athletes at the Talent stage, this effect was not statistically significant among elite athletes at the Elite stage, although this finding may be inconclusive due to the small sample size (Table 7). Third, the logistic regression analysis indicated significant predictors of elite status change across developmental stage (Table 8). Specifically, sex (female), sports transitioning (yes), and the 20m-shuttle run score were consistently emerged as significant predictors across most grades, while body mass only significant in 8th and 9th grades. No other anthropometric or physical fitness measures were not predictive at any grade level.

Taken together, within this particular L-TID cohort (i.e., composed of individuals with superior physical fitness) demonstrated that sex (female) and sports transitioning exhibited a stronger statistical association with elite status within this specific cohort than early physical advantages or RAE. These results highlight the potential role of structural and developmental factors within this L-TID environment in predicting future elite progression, though these associations require further verification.

### 4.1 Sports transitioning

The cgs sports investigated in this study (i.e., sports measured in centimeters, grams, or seconds) are characterised by a strong emphasis on physical capabilities and relatively lower technical and tactical requirements (28). This sports category is conceptually consistent with the notion of “late specialisation”, as it contrasts with early specialisation (e.g., gymnastics). Empirical evidence supports this view, indicating that intensive training adaptations during adolescence are crucial for attaining long-term athletic success (28, 29).

Elite athletes in this cohort achieved success primarily in a limited number of sports, with many reaching that level through “sports transition”. While logistic regression identified sports transitioning as a predictor of elite attainment in this study, this should be interpreted as a context-specific association within the Fukuoka L-TID programme rather than definitive evidence of deliberate optimal matching. This finding aligns with the value of diverse sporting experiences in childhood as a foundation for later success. In this context, sports transitioning should be viewed as a context-specific association where accumulated experiences across multiple sports could be leveraged to support long-term development.

Most elite athletes in this study transitioned to late-specialisation sports such as athletics, cycling, and rowing. Moesch et al. (28) argued that late specialization is advantageous in cgs sports, where physical and mental maturity are critical. It is likely that these athletes optimised their potential by gaining broad experiences early by focusing on their main sport later. Importantly, the higher success rate observed in these sports may reflect structural advantages, such as lower national participation density or reduced selection barriers for senior representation, rather than the superiority of the programme's matching mechanisms alone.

Moreover, the concentration of elite athletes in certain sports suggests that these sports benefitted from robust regional development environments. As emphasised by Henriksen & Stambulova (30), a supportive social context including family, schools, clubs, and federations likely functioned as a comprehensive development environment for elite success. It can also be inferred that these sports maintained well-established cooperative relationship with their National Federations (NFs). As De Bosscher & De Rycke (31) reported, the effectiveness of system-level talent development programmes heavily depends on collaboration with national governing bodies. Such partnerships likely facilitated structured athlete development pathways that supported talented individuals reaching elite status. Taken together, these findings indicate that in the specific sports where athletes achieved elite status in the L-TID case study, their success was supported by three key factors: diverse sporting experiences during childhood, late specialisation, and strong collaboration between the regional development environments and the NFs.

### 4.2 RAE patterns

Our study demonstrates a strong RAE among L-TID graduates at the Talent stage, but this effect appears to diminish among senior elite athletes (Table 7). Furthermore, RAE was not identified as a predictive factor in logistic regression analysis (Table 8). This pattern is consistent with previous findings suggesting that RAE varies across developmental stages (32, 33). The pronounced RAE pattern observed at the Talent stage supports the widely accepted view that, within a given age group, relatively older children tend to be more physically mature and fit, giving them a selection advantage (32–35). In contrast, the diminished RAE among senior elite athletes suggests that the phenomenon does not persist throughout long-term development, a finding corroborated by several international researchers. For example, Kelly et al. (36) found that in men's rugby union, early-born athletes were overrepresented in youth academies, but this effect disappeared at the professional and national levels. Similarly, Brustio et al. (37) observed that in athletics, RAE was evident in the U18 category but absent at the senior level, with relatively younger athletes more likely to successfully transition to the senior elite stage. However, it must be acknowledged that the small elite sample size (*n* = 31) limited statistical power of the analysis, meaning that the presence of a small-to-moderate RAE effect cannot be definitively rule out.

Furthermore, the persistence and direction of RAE patterns can vary by sport and even by position. Jones et al. (38) reported a traditional RAE among elite cricket batters and rugby backs, but a reverse trend among rugby forwards, where late-born athletes were overrepresented. No RAE was found for cricket pace bowlers. These findings suggest that physical attributes that confer early advantages may become less critical at the elite level, where technical skills, tactical understanding, and psychological resilience become more decisive (35). Moreover, Sasano et al. (19) reported that highly skilled athletes may overcome initial selection biases during their development. As Mann & van Ginneken (33) suggested, the transparent presentation of athletes' birth-month data during junior selection processes could help mitigate RAE-related biases. Taken together, the findings from the L-TID case study indicate that while RAE strongly influences initial selection at the Talent stage, its effect diminishes or disappears among senior elite athletes who progress through athlete development pathways. Consequently, while RAE did not emerge as a significant predictor among senior elite athletes in this study, this finding remains inconclusive due to the limited statistical power of the small elite sample. Therefore, RAE should not be definitively ruled out as a potential influence on long-term development.

### 4.3 Consistent predictors of elite status

The present study identified two consistent predictors of elite attainment from the Talent and Elite stages: sex (female) and sports transitioning (Table 8). Notably, being female showed a significant statistical association with elite status within this cohort. However, the exceptionally high *OR*s (e.g., 127.696 in 9th grade) and the wide 95% *CI*s for these estimates likely reflect statistical instability and uncertainty due to the small elite sample size and cohort imbalance. Therefore, these findings should be interpreted as programme-specific observations rather than generalized trends. Sports transitioning also maintained a significant and positive effect across all models, with high *ORs* observed in the upper elementary grades (e.g., 27.047 in 5th grade; 7.140 in 6th grade).

The strong association between female athletes and elite attainment within the L-TID programme (Table 8) is particularly significant when contrasted with well-documented challenges surrounding female sport engagement and retention in Japan. The decline in sport and physical activity participation among females becomes particularly evident during the transition between educational stages (25). For example, the time spent on physical activity outside compulsory physical education classes decreases significantly from upper elementary to junior high school. Consequently, the sport participation rate among females drops sharply in their 20s–40s (39). Data from national sport organizations also reveal that the gender disparity in participation rates, already present in youth categories, significantly widens in adulthood (40, 41). This cumulative decline represents a substantial talent loss in the female athlete talent pipeline during adolescence.

The reasons behind this female disengagement from sport participation are multifaceted, rooted in socio-cultural factors and systemic issues within the sporting environment. Previous study indicated that barriers such as aversion to sun exposure, discomfort from sweat or fatigue, and fear of negative social evaluation, or competition/ranking (42). In addition, limited understanding among coaches of female-specific developmental and physiological factors contribute to higher dropout rates (43, 44). Surveys consistently show that women report greater feelings of exercise deficiency than men across multiple age groups, highlighting a broader public health concern (45).

Therefore, the exceptionally high *OR*s observed for females (e.g., *OR* = 127.696 in 9th grade) are best interpreted as the result of a favorable confluence between the external environmental factors within the Japanese sporting context (i.e., the current status of female sport participation and retention.) and the programme's structural factors (e.g., talent selection concept, multi-sport strategy, and coach/guardian's competency, etc.). While the programme's structure is designed to offer equivalent opportunities to both sexes, this design appears to have mitigated some structural and socio-cultural barriers (e.g., coaching practices, societal expectations) that typically lead to high dropout rates among female athletes in Japan. Within the specific context of this programme, this association suggests it may be particularly beneficial for talented female athletes, enabling their continued participation and progression to elite status by addressing the gender-specific challenges prevalent in the Japanese sports environment.

The consistent and strong positive predictive relationship between sporting history and talent identification reinforces the programme's emphasis on non-early specialization (Table 8). This approach aligns with established Long-Term Athlete Development models advocating for multi-sport participation in early years to build a broad movement skill foundation and enhance physical literacy (11, 46). The exceptionally high *OR*s observed in upper elementary grades (e.g., *OR* = 27.047 in 5th grade) suggest that early exposure to diverse sports prior to specialisation is a critical foundation for developing high-level athletic potential. Such diversity enhances adaptability, reduces the risk of overuse injuries and burnout associated with early specialisation (47), and subsequently supports athletes' natural alignment with sports best suited to their matured physical and psychological profiles. The TID programme's capacity to identify late-emerging talent through sports transitioning, rather than favoring early performers, represents a key structural strength that maximises the potential of overall athlete pool.

### 4.4 Limitation

This study has several limitations. First, the limited sample size of the elite athlete group (*n* = 31) resulted in wide 95% *CI*s for the key *OR*s, leading to residual uncertainty in the precision of these estimations. While the consistent direction and significance of the *OR*s across grades offer strong evidence, future studies with larger cohorts are necessary to enhance the precision of these results. Furthermore, the low number of athletes who successfully transitioned from the L-TID programme to senior elite representation, along with the fact that this study is based on a case study from a single region (prefecture), limits the generalizability of our findings (48, 49). However, the current findings are valuable for providing new perspectives to L-TID practitioners in comparable settings.

Second, although we focused on anthropometric and fitness characteristics, sports performance is also influenced by psychological skills, technical proficiency, and tactical awareness (50, 51). Thus, future research should adopt a more holistic, multidisciplinary approach than what currently exists (49, 52).

Third, identifying and developing talent among elementary and junior high school athletes remains inherently challenging task, as long-term success at the Talent stage is difficult to predict, raising questions about the cost-effectiveness of such programmes (53–56). However, within the context of Japanese sporting culture, educational and personal-development outcomes such as life skills, interpersonal skills, and social competence may justify these TID programmes beyond performance outcomes.

Furthermore, extrapolation of these findings to other geographical regions or TID systems requires verified application that explicitly considers differences in sport composition and institutional design. Future research should explore how cultural contexts and diverse of TID philosophies across nations shape the broader objectives and roles of these programmes. It is also important to examine how specific experiences within TID programmes influence the long-term performance trajectories of elite athletes.

### 4.5 Practical recommendations

Based on these findings, the following recommendations are proposed for the L-TID practitioners and broader policy makers:

Recommend support for female athletes and sports transitioning: Given their significant influence across all developmental stages, future strategies should prioritise structural support for female athlete retention and integrate initiatives that promote multi-sport experience and deliberate talent transfer/optimisation.
Adopt maturation-adjusted evaluation criteria: From junior high school onward (8th grade and above), where body mass has emerged as a significant predictor, evaluation should incorporate maturation-adjusted metrics to ensure equitable assessments of potential that recognise developmental variability rather than relying on size-dependent performance measures.
Shift from predictive screening to holistic development: TID should move beyond reliance on RAE or general physical fitness as primary indicators. Instead, programmes need to adopt a holistic developmental approach that considers the regional and developmental pathways of elite success and nurtures the interplay of diverse sporting experiences, psychological skills (not measured in this study), and adaptability, particularly during the Talent stage.
Future paradigm shift and generalisation: This study, serving as a critical pilot investigation, suggests that within the Japanese L-TID system, prioritizing organisational design and strategic developmental pathways over initial performance metrics may be beneficial. While the generalisability of these findings remains limited to similar sporting environments, the evidence within this specific context supports a focus on strategic development that considers institutional and cultural differences.

## 5 Conclusions

This case study, based on a longitudinal analysis of the Fukuoka TID cohort selected for superior athletic ability, identified the key structural and developmental characteristics that distinguish youth who successfully transition to the senior elite level.

We identified a significant statistical association between structural/developmental factors and elite attainment in this cohort. Specifically, being female and having experience in sports transitioning were the most consistent predictors observed, though the magnitude of these effects may be specific to this programme's context.

Taken together, these results highlight the importance of institutional design and planned developmental history. These findings suggest that within this specific L-TID model, structural factors show a stronger association with long-term success than initial physical advantages. While these structural factors were the strongest predictors in this study, future programmes are required to adopt a truly holistic approach (one that integrates unmeasured elements like psychological skills and adaptability) to ensure sustained elite success.

## Data Availability

The datasets presented in this article are not readily available because data sharing for this study is restricted due to ethical reasons and the specific terms of approval granted by the first author's Institute Ethics Committee, which stipulated that data must be strictly managed and not shared with third parties. However, requests for data sharing may be considered by the corresponding author and the granting institution, provided there is a scientifically and ethically justifiable reason, in compliance with institutional guidelines. Requests to access the datasets should be directed to Masahiro Hagiwara: masahiro.hagiwara@jpnsport.go.jp.
